# Life without double-headed non-muscle myosin II motor proteins

**DOI:** 10.3389/fchem.2014.00045

**Published:** 2014-07-07

**Authors:** Venkaiah Betapudi

**Affiliations:** ^1^Department of Cellular and Molecular Medicine, Lerner Research Institute, Cleveland ClinicCleveland, OH, USA; ^2^Department of Physiology and Biophysics, Case Western Reserve UniversityCleveland, OH, USA

**Keywords:** myosin II, motor proteins, molecular machines, cell migration, cytokinesis, cancer, pathogenesis, microparticles

## Abstract

Non-muscle myosin II motor proteins (myosin IIA, myosin IIB, and myosin IIC) belong to a class of molecular motor proteins that are known to transduce cellular free-energy into biological work more efficiently than man-made combustion engines. Nature has given a single myosin II motor protein for lower eukaryotes and multiple for mammals but none for plants in order to provide impetus for their life. These specialized nanomachines drive cellular activities necessary for embryogenesis, organogenesis, and immunity. However, these multifunctional myosin II motor proteins are believed to go awry due to unknown reasons and contribute for the onset and progression of many autosomal-dominant disorders, cataract, deafness, infertility, cancer, kidney, neuronal, and inflammatory diseases. Many pathogens like HIV, Dengue, hepatitis C, and Lymphoma viruses as well as *Salmonella* and *Mycobacteria* are now known to take hostage of these dedicated myosin II motor proteins for their efficient pathogenesis. Even after four decades since their discovery, we still have a limited knowledge of how these motor proteins drive cell migration and cytokinesis. We need to enrich our current knowledge on these fundamental cellular processes and develop novel therapeutic strategies to fix mutated myosin II motor proteins in pathological conditions. This is the time to think how to relieve the hijacked myosins from pathogens in order to provide a renewed impetus for patients' life. Understanding how to steer these molecular motors in proliferating and differentiating stem cells will improve stem cell based-therapeutics development. Given the plethora of cellular activities non-muscle myosin motor proteins are involved in, their importance is apparent for human life.

## Introduction

Machines are involved in driving virtually every aspect of modern human life, and so are myosin motor proteins in driving cellular life. Myosin motor proteins are specialized molecular machines that convert cellular free-energy into mechanical work (Bustamante et al., 2004). It is largely believed that the myosin-performed mechanical work intersects with almost every facet of cell biology. In fact, myosins play a central role in driving cellular activities that are necessary for singing a courtship song in flies, reproduction, childbirth, growth, development, and immunity as well as predisposing humans to a certain degree of risk for diseases (Stedman et al., 2004; Maravillas-Montero and Santos-Argumedo, 2012; Slonska et al., 2012; Chakravorty et al., 2014; Min et al., 2014; Pecci et al., 2014).

The biological cell is equipped with a wide variety of motor proteins that are divided into cytoskeletal (myosin, kinesin, dynein), polymerization (actin, microtubule, dynamin), rotary (F_0_F_1_-ATP synthase), and nucleic acid (RNA and DNA polymerases, Helicase, Topoisomerases, RSC, SW1/SNF complex, SMC, viral DNA packaging protein) motor proteins to perform specific and dedicated cellular functions (Kolomeisky, 2013; Howard, 2014). Interestingly, these specialized molecular machines not only operate in a world where Brownian motion and viscous forces dominate but also work more efficiently than man-made combustion engines (van den Heuvel et al., 2007; Kabir et al., 2011). No biological cell can operate in the absence of these molecular machines. Most of these motor proteins are ubiquitously expressed but the expression of some of these motor proteins depends on cell and tissue type. The present review is about myosin motor protein, an essential component of the cytoskeletal system that is made up of proteins encoded by 441 genes in human. The human genome contains 40 genes that encode myosin motor proteins.

The term “myosin” (myo- + -ose + -in) means within muscle and was used to describe proteins with ATPase activity found originally in striated and smooth muscle cells (Pollard and Korn, 1973). The term “myo” was originated from “mys” to denote muscle in Greek. More than 140 myosins are reported in eukaryotes except in red algae and diplomonad protists (Vale, 2003). The majority of myosins have distinct head, neck, and tail domains and they are categorized into 35 different classes based on phylogenic analysis of their conserved heads, domain architectures, specific amino acid polymorphisms, and organismal distributions (Richards and Cavalier-Smith, 2005; Foth et al., 2006; Odronitz et al., 2007). Each class of myosins received a roman numeral. If more than one myosin of the same class is expressed in an organism, they are named in an alphabetical order according to their discovery. The present review is focused on current understanding and recent advances in various aspects of selected class II myosins as well as their regulation and relevance to human life and diseases.

## Class II myosins (myosin II)

**Figure 1:**
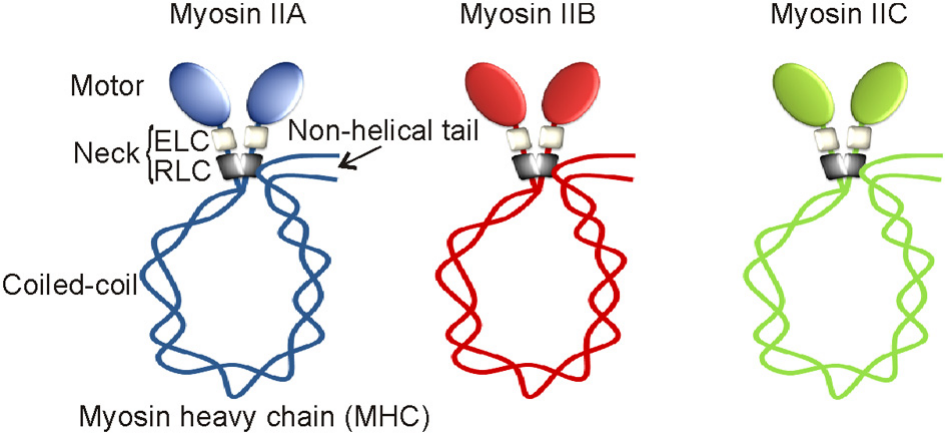
**Non-muscle myosin II motor proteins**. Schematic representation of myosin II motor proteins that exist as complexes in cells.

More than seven decades ago, an unknown myosin with ATPase activity was reported in the extracts of muscles (Engelhardt and Liubimova, 1994). Later, that unknown muscle myosin was identified as a class II myosin and then called conventional myosin and or the founding member of myosin super family. Class II myosins are expressed in all eukaryotes except plants. More than 34 class II myosins are reported in different organisms to date (Bagshaw, 1993). At least one myosin II is believed to be expressed in all eukaryotic cells. Based on motor or tail domain sequences and cell type expressions, class II myosins are further divided into four different sub-classes or groups. They are (1) *Acanthamoeba* or *Dictyostelium* myosins, (2) yeast myosins, (3) skeletal or cardiac or sarcomeric myosins, and (4) vertebrate smooth muscle or non-muscle myosins. Class II myosins are believed to be originated in unikonts that are ancestral eukaryotes with or without a single flagellum, including amoebozoans, fungi, and holozoans (Richards and Cavalier-Smith, 2005). While simple unicellular organisms like amoeba adopted a single myosin II gene, complex multicellular organisms except *Drosophila* acquired multiples of them during evolution. The human genome has over 40 myosin genes, and 15 of them are class II myosin genes (*MYH1, MYH2, MYH3, MYH4, MYH6, MYH7, MYH7B, MYH8, MYH9, MYH10, MYH11, MYH13, MYH14, MYH15, MYH16*) but not all of them are active (Berg et al., 2001). *MYH11* encodes myosin II in smooth muscles but its splice variants result in four distinct isoforms (Matsuoka et al., 1993). *MYH9, MYH10*, and *MYH14* located on different chromosomes encode myosin IIA, myosin IIB, and myosin IIC, respectively (Figure 1). These myosin II motor proteins are expressed exclusively in non-muscle cells, therefore called non-muscle myosin II motor proteins (Simons et al., 1991; Toothaker et al., 1991; Leal et al., 2003; Golomb et al., 2004). Myosin IIA, myosin IIB, and myosin IIC are expressed in every human non-muscle cell with a few exceptions; however, their expressions depend on cell and tissue types (Kawamoto and Adelstein, 1991; Golomb et al., 2004). No tissue or cell type appears to express all three non-muscle myosin II motor proteins but many cell types express at least one or two of them under normal physiological conditions. Myosin IIA and myosin IIB are expressed in endothelial and epithelial cells at similar levels. However, myosin IIB and myosin IIC are expressed abundantly in nervous and lung tissue, respectively. Myosin IIA is the only conventional myosin II motor protein expressed in the circulating platelets. Thus, preferential expression of myosin II motor proteins in different cell types reflects their specialization in mediating separate, dedicated, and probably non-redundant cellular functions. Why doesn't a single cell or tissue type express all three myosin II motor proteins is yet to be clearly understood. Perhaps, the cell specific expression of myosin II paralogs is critical for maintaining different cell and tissue types.

Myosin II motor proteins are mostly found in the cytoplasm of quiescent cells except in the nuclei of proliferating myoblasts (Rodgers, 2005). The cytosolic myosin II motor proteins undergo transient localization to contractile ring during cytokinesis. Myosin II motor protein using ATP as a cytosolic fuel generates mechanical forces required for separation of daughter cells during cytokinesis. However, the specific roles and underlying mechanisms of myosin II paralogs during cytokinesis are not clearly understood. The functional and mechanical roles of non-muscle myosin II motor proteins are extensively investigated in migrating cells for the past two decades. Many laboratories reported myosin IIA and myosin IIB with specific roles in mediating cell shape changes and interaction with matrix during migration. Cells prefer to make periodic extension and retraction of their lamellipodia during migration by unknown mechanisms. Interestingly, myosin IIA and myosin IIB motor proteins localize distinctly in the lamellipodia of migrating cells. On one hand, myosin IIB promotes lamellipodia and growth cone extensions and on the other, myosin IIA drives retraction of cell membrane during cell migration (Rochlin et al., 1995; Brown and Bridgman, 2003; Betapudi, 2010). The specific roles of myosin IIC motor protein in driving cell migration are not clearly understood. Myosin II activity is necessary for keratinocytes' migration, a critical step in the re-epithelialization of human skin wound (Betapudi et al., 2010). Myosin II motor proteins are also required for internalization of the cell surface receptors including EGFR and CXCR4 (Rey et al., 2007; Kim et al., 2012). Myosin II-mediated mechanical forces have been implicated in operating the activity of contractile vacuoles to expel additional water and toxic materials from the soil-living amoeba in hypo-osmotic conditions (Betapudi and Egelhoff, 2009). Myosin II motor proteins have also been implicated in the mediation of viral infection (van et al., 2002; Arii et al., 2010), microparticle secretion (Betapudi et al., 2010), and cell death (Solinet and Vitale, 2008; Flynn and Helfman, 2010; Tang et al., 2011), however, their specific roles and underlying mechanisms remain unclear. Lower eukaryotes, such as amoeba can survive with certain developmental defects in the absence of myosin II (Xu et al., 1996) but the expression of all three myosin II motor proteins are necessary for mouse embryo growth and development (Conti and Adelstein, 2008).

## Non-muscle myosin II complex

In line with multiple components involved in the assembly of man-made machines, biological cells also build their molecular machines using multiple polypeptides that are encoded by different genes. For instance, myosin II motor protein exists as a complex consisting of six non-covalently associated polypeptides that are encoded by a single myosin II and two different non-myosin genes. Each myosin II complex with 525 kDa molecular weight is composed of a myosin II heavy chain (MHC) homodimer, two essential light chains (ELC), and two regulatory light chains (RLC). Based on their extraction methods, ELC and RLC are also called alkali and 5,5'-dithiobis/2-nitrobenzoate (DTNB) light chains, respectively. While MHC with 226 kDa molecular weight is encoded by a myosin II gene, both ELC with 16 kDa and RLC with 22 kDa molecular weights are considered as non-myosin proteins of myosin II complex. Both ELC and RLC are commonly found in all myosin II complexes. Alternatively spliced MHC, ELC, and RLC are known to be expressed in certain tissue but our current knowledge on their specificities is still limited. Both heavy and light chain peptides undergo the UCS (UNC-45/Cro1/She4) chaperone-mediated proper folding and assembly regulation in order to form a functional myosin II complex in the Golgi apparatus (Gazda et al., 2013; Hellerschmied and Clausen, 2013). This understudied complicated assembly process is common for all three myosin II motor proteins remains elusive. Transcriptional regulations of ELC and RLC are not clearly understood; however, MHC expressions of all three myosin II motor proteins are under the control of house-keeping promoters having no TATA elements (Kawamoto, 1994; Weir and Chen, 1996). However, differential expressions of MHCs were observed in response to serum and mitotic stimulants (Kawamoto and Adelstein, 1991; Toothaker et al., 1991). Elevated levels of MHCs were found in many types of tumor tissues (our unpublished results) but their underlying mechanisms are not clearly understood.

The MHC of class II myosins can be subdivided into distinct head, neck, and tail functional domains. Except C-terminal tail pieces, the MHCs of myosin IIA, myosin IIB, and myosin IIC share a significant protein sequence similarity in their motor domains. The N-terminal catalytic globular head or motor domain has binding sites for actin and ATP. Motor domain is also called the functional engine of myosin II motor protein. Myosin II motor domain undergoes an ATP-dependent conformational change in order to control its interaction with actin filaments, a key element of the cell strategy to convert cellular free-energy into protein motion or mechanical work. Despite having a significant sequence similarity, myosin II motor domains carry different binding affinities for actin filaments. Thus, myosin IIA, IIB, and IIC are believed to perform mechanical work with different energetic efficiencies in cells. Myosin II motor domain is followed by a neck region consisting of two conserved IQ motifs (IQxxxRGxxxR); however, myosins of other classes may have more or less than two IQ motifs (Cheney and Mooseker, 1992). IQ motifs form an amphiphilic uninterrupted seven-turn α-helix with binding affinity for either light chains or calmodulin in Ca^+2^-independent manner. ELC and RLC occupy the first and second IQ motifs of the neck region, respectively. ELC binds IQ motif to give stability for MHC; however, RLC offers both stability and functional regulation to MHC. IQ domain allows light chains to acquire either compact or extended conformation. Thus, neck region with light chains attached acts as a linker and lever arm for myosin II motor domain to amplify energy conversion into mechanical work. The length of the neck region is believed to have direct impact on myosin II motor speed and energy transduction into mechanical work (Uyeda et al., 1996). The neck region of all myosins have IQ motifs except class XIV Toxoplasma myosin A (Heintzelman and Schwartzman, 1997). IQ motif with approximately 25 amino acids in length is widely distributed in nature, thus, ELC also binds other myosins of class V, VI, and VII as well as non-myosin proteins carrying IQ motifs, but RLC exclusively binds to myosins of class II and XVIII (Chen et al., 2007; Tan et al., 2008). Myosin II neck region is followed by a tail domain with variable amino acid sequences. The tail domain with coiled-coil α-helices terminates into a short non-helical tailpiece. The coiled-coil tail domain undergoes homodimerization to form a single rod-like structure. Thus, myosin II complex has two globular heads or motor domains with a single coiled-coil rod-like structure hence called double-headed myosin II motor protein. Myosin II complex attains a compact folded conformation due to a “proline-kink” at the junction of head and rod domains, and attachment of its C-terminal tail domain to RLC as depicted in Figure 1 (Onishi and Wakabayashi, 1982; Trybus et al., 1982; Craig et al., 1983). Thus, the myosin II complex with compact folded structure sediments at 10 S (Svedberg) and therefore called 10S form. The myosin II complex in 10S form shows high binding affinity for ADP and inorganic phosphate (Pi), and virtually no enzyme activity (Cross et al., 1986, 1988). However, the activated myosin II complex exists in an elongated conformation due to its C-terminal tail detachment from RLC. The activated myosin II complex in an elongated form sediments at 6 S and therefore called 6S form (Trybus and Lowey, 1984). Myosin II motor proteins with elongated conformation tend to assemble into highly ordered parallel and anti-parallel thick filaments due to intermolecular interactions between coiled-coil tail domains. Interestingly, myosin II tail domains form large aggregates without proper filamentation in the absence of RLC (Pastra-Landis and Lowey, 1986; Rottbauer et al., 2006). Thus, RLC-controlled tail-domain filamentation and motor domain interaction with actin filaments are the most important aspects of cell strategy for converting ATP released free-energy into force and mechanical work using myosin II motor proteins.

## RLC phosphorylation in regulating myosin II activity

**Figure 2:**
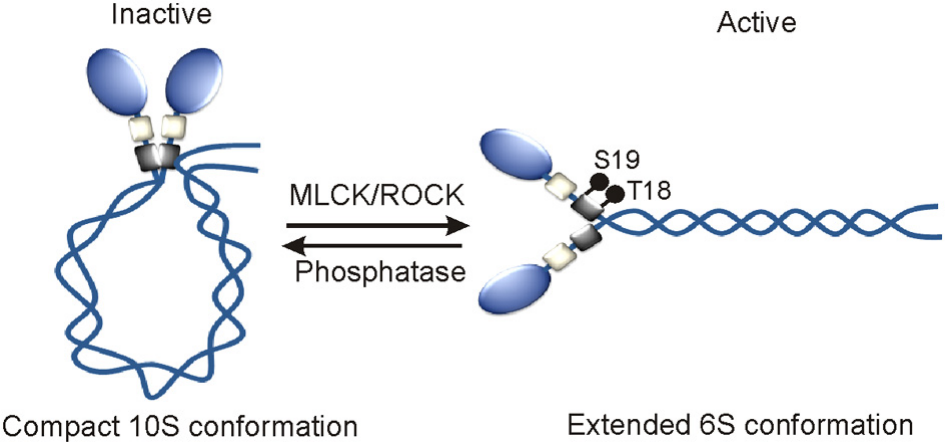
**Mechanism of the activation of myosin II motor proteins**. RLC phosphorylation by MLCK and ROCK or other kinases turns on myosin II motor protein *in vivo*.

Myosin IIA, myosin IIB, and myosin IIC paralogs with 60–80% sequence similarity at the amino acid level and same quaternary structure appear to be diverged from a common ancestor more than 600 million years ago, however, they display different regulatory mechanisms under normal physiological conditions (Jung et al., 2008). Role of RLC phosphorylation in regulating myosin II activity in many cell and tissue types is extensively investigated since its discovery in rabbit skeletal muscle myosins more than three decades ago (Casadei et al., 1984). RLC perhaps does not exist alone but when remains associated with the neck region of MHC undergoes reversible phosphorylation on its S1, S2, T9, T18, and S19 amino acids in order to turn-on and turn-off myosin II motor complexes in cells (Figure 2). RLC phosphorylation on S19 alone or on both T18 and S19 amino acids turns-on myosin II motor complex by increasing its ATPase activity and extended 6S conformation that allows simultaneous assembly into thick filaments (Wendt et al., 2001; Somlyo and Somlyo, 2003; Betapudi et al., 2006, 2010). However, RLC phosphorylation does not affect myosin II motor domain affinity for actin filaments (Sellers et al., 1982). RLC phosphorylation on S1, S2, and S9 or dephosphorylation on T18 and S19 amino acids turns-off myosin II complex by allowing acquisition of monomeric 10S compact conformation and no filamentation.

RLC reversible phosphorylation is tightly regulated by both myosin specific phosphatase and a wide variety of kinases including myosin light chain kinase (MLCK/MYLK), Rho-associated coiled-coil-containing kinase (ROCK), leucine zipper interacting protein kinase (ZIPK) or death associated protein kinase 3 (DAPK3), citron kinase or citron rho-interactive kinase (CRIK) or Serine/threonine-protein kinase 21 (STK21), myotonic dystrophy kinase-related CDC42-binding kinase (MRCK/CDC42BP). These kinases are known to phosphorylate RLC on T18 and S19 amino acids to activate myosin II complexes in different cell types. Protein kinase C (PKC) phosphorylates S1, S2, and S3 amino acids to inactivate myosin II in dividing cells (Nishikawa et al., 1984; Varlamova et al., 2001; Beach et al., 2011). Interestingly, all these kinases display specific intracellular localizations and respond to a wide variety of signal transduction pathways in order to phosphorylate RLC and activate myosin II motor proteins in many cell types. MLCK in response to Ca^+2^-calmodulin activates myosin II that is localized next to cell membrane (Totsukawa et al., 2004). The site-specific intracellular localization and activity of MLCK are regulated by several kinases including p21 activated kinase 1 (PAK1), Abl tyrosine kinase, Src, and arrest defective 1 in many cell types (Sanders et al., 1999; Dudek et al., 2004; Shin et al., 2008). RhoA, a small GTP-binding protein activates both ROCK and citron kinase in the central part of cell. The actin binding protein, Shroom3 directs ROCK intracellular localization, and RLC phosphorylation in neuroepithelial cells (Haigo et al., 2003; Hildebrand, 2005). DAPK3 predominantly displays nuclear localization and phosphorylates RLC in the cells that are undergoing apoptosis in a Ca^2+^/calmodulin-independent manner (Murata-Hori et al., 1999). PKC phosphorylates RLC in the presence of Ca^+2^ and DAG (diacylglycerol) and or phorbol esters in mitotic cells (Varlamova et al., 2001). Both intracellular site-specific RLC reversible phosphorylation and myosin II activation are tightly controlled by protein phosphatase 1 (PP1), a ubiquitously expressed myosin specific phosphatase (Xia et al., 2005; Matsumura and Hartshorne, 2008; Rai and Egelhoff, 2011). All the regulators of RLC phosphorylation are also known to phosphorylate other substrates in cells. For instance, MLCK is implicated in phosphorylating a proline-rich protein tyrosine kinase 2 (PYK2/PTK2B) or focal adhesion kinase 2 (FAK2) that is involved in promoting lung vascular endothelial cell permeability during sepsis (Xu et al., 2008). ROCK also directly phosphorylates LIM kinase and MYPT1, a regulatory subunit of PP1 in many types of cells and tissues (Kimura et al., 1996; Leung et al., 1996). MYPT1 phosphorylation inactivates PP1 and this leads to a marked increase in RLC phosphorylation and myosin II activation. MYPT1 phosphorylation is also regulated by ZIPK, MRCK, and PKC in many cell and tissue types. PKC also phosphorylates MHC to regulate myosin II activity in cells under normal physiological conditions. MLCK-A is the only RLC phosphorylating kinase identified in *Dictyostelium* to date (Tan and Spudich, 1990). Unlike MLCK in mammalian cells, MLCK-A phosphorylates S13 of RLC in the absence of Ca^+2^-calmodulin (Tan and Spudich, 1990). The RLC phosphorylation on S13 amino acid increases myosin II motor activity and regulates cell morphological changes without affecting normal growth and development of amoeba (Griffith et al., 1987; Chen et al., 1994; Uyeda et al., 1996; Liu et al., 1998; Matsumura, 2005). Except reversible phosphorylation, no other posttranslational modification of RLC that has a role in regulating myosin II activity is known to date.

## MHC phosphorylation in regulating myosin II activity

**Figure 3:**
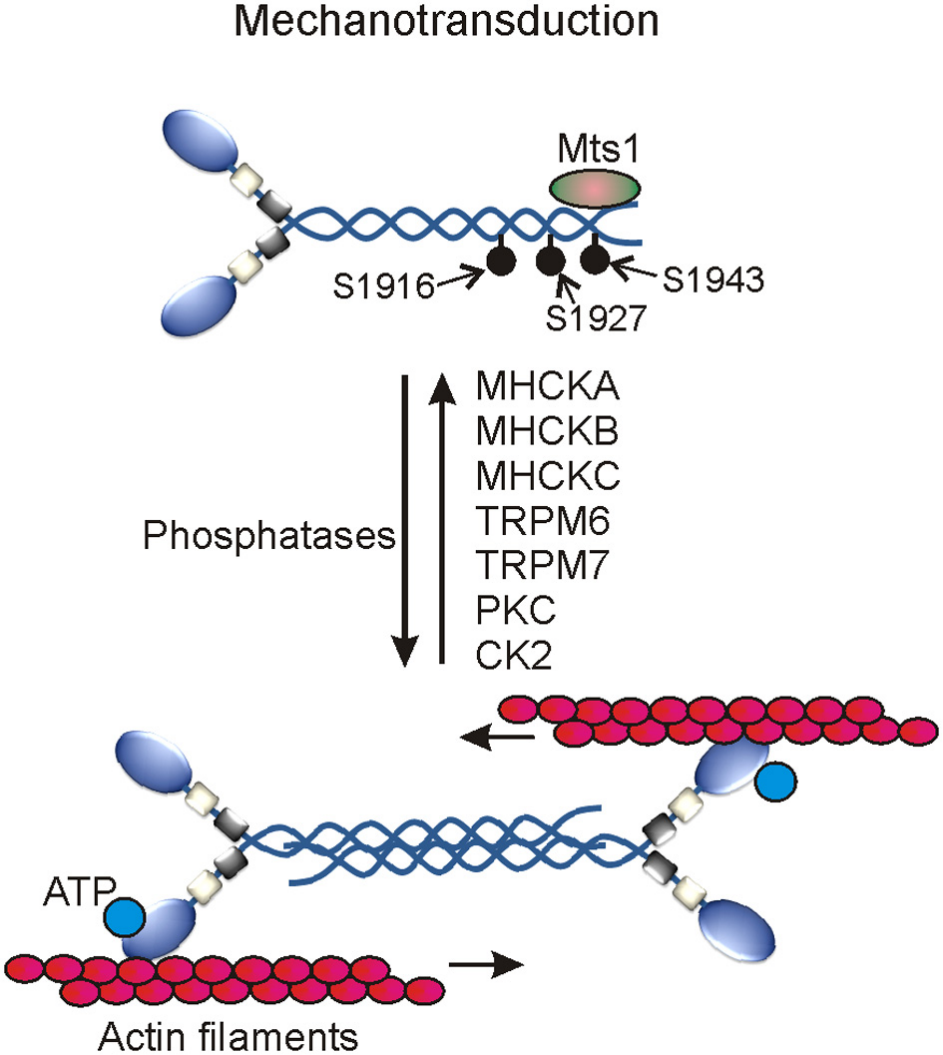
**Myosin II motor proteins-mediated mechanotransduction in cells**. Several myosin II heavy chain specific protein kinases activate myosin II motor proteins. The activated myosin II associates with actin filaments to generate contractile forces using cellular ATP.

MHC phosphorylation was first reported in macrophages in the early 1980s and after nearly a decade its role in regulating myosin II filamentation and localization was documented in lower eukaryotes like *Acanthamoeba* and *Dictyostelium disoideum* (Collins and Korn, 1980; Kuczmarski and Spudich, 1980; Trotter, 1982; Kuznicki et al., 1983; Trotter et al., 1985; Barylko et al., 1986; Pasternak et al., 1989; Egelhoff et al., 1993). According to computational prediction of phosphorylation sites, the heavy chains of myosin IIA, IIB, and IIC appear to undergo phosphorylation on multiple residues in the head, neck, and tail domains; however, only a few sites in the coiled-coil and non-helical tail regions of their C-terminal ends are reported to date. The MHC of myosin IIA undergoes phosphorylation on T1800, S1803, and S1808 in the coiled-coil and on S1943 residues in the non-helical tail regions (Figure 3). Myosin IIB and myosin IIC heavy chains also undergo phosphorylation on multiple sites in the coiled-coil and non-helical tail regions of their C-terminal ends (Dulyaninova and Bresnick, 2013). Many kinases including casein kinase 2 (CK2), the members of PKC as well as alpha-kinase family are involved in phosphorylating C-terminal ends of all three MHCs in normal physiological and pathological conditions (Murakami et al., 1998; Dulyaninova et al., 2005; Clark et al., 2008a,b; Ronen and Ravid, 2009). PKC members are involved in phosphorylating S1916 and S1937 residues of myosin IIA and myosin IIB, respectively (Conti et al., 1991; Even-Faitelson and Ravid, 2006). PKC is also involved in phosphorylating other multiple serine residues in myosin IIB and threonine residues in myosin IIC coiled-coil regions (Murakami et al., 1998; Ronen and Ravid, 2009). CK2 is known to phosphorylate S1943 residue in the non-helical tail region of myosin IIA *in vitro*. CK2 was implicated in the regulation of myosin II assembly and localization especially in pathological conditions. However, neither chemical inhibition nor siRNA-mediated depletion of CK2 showed any effect on S1943 phosphorylation or breast cancer cell migration on fibronectin coated surfaces (Betapudi et al., 2011). CK2 is also involved in phosphorylating multiple residues in the coiled-coil and non-helical tail regions of myosin IIB and myosin IIC (Murakami et al., 1998; Ronen and Ravid, 2009; Rosenberg et al., 2013). Thus, CK2 clearly plays a critical role in regulating myosin II-mediated cellular functions in other pathological conditions.

In addition to PKC and CK2, several members of the alpha-kinase family are involved in phosphorylating myosin II heavy chains in mammals and *Dictyostelium discoideum*. Alpha kinases belong to a small and unique group of protein kinases with catalytic domains having a little or no similarity at amino acid level with the catalytic domains of conventional protein kinases (Ryazanov et al., 1999; De la Roche et al., 2002; Drennan and Ryazanov, 2004; Scheeff and Bourne, 2005; Middelbeek et al., 2010). Conventional protein kinases usually find their phosphorylating sites in β-turns, loops, and irregular structures of their substrates; however, the first member of the alpha-kinase family prefers to phosphorylate amino acids located in the α-turns of their cellular targets hence called α-kinases (Vaillancourt et al., 1988; Luck-Vielmetter et al., 1990). But recent *in vitro* phosphorylation studies showed that alpha-kinases also target residues present in the non-alpha helical structures of their cellular substrates (Jorgensen et al., 2003; Clark et al., 2008a). Members of the alpha-kinase family are identified only in eukaryotes to date (Ryazanov et al., 1999; Scheeff and Bourne, 2005). Transient receptor potential melastatin 6 (TRPM6) and Transient receptor potential melastatin 7 (TRPM7) kinases are among the total six alpha-kinases identified in human to date. TRPM6 and TRPM7 kinases belong to a large protein family of transient receptor potential cation channels that are involved in sensing mechanical stress, pain, temperature, taste, touch, and osmolarity (Ramsey et al., 2006; Middelbeek et al., 2010; Su et al., 2010; Runnels, 2011; Mene et al., 2013). Both TRPM6 and TRPM7 kinases phosphorylate T1800, S1803, and S1808 residues in the coiled-coil region of MHC to control myosin IIA filamentation and association with actin filaments (Clark et al., 2008a,b). These multifunctional kinases also phosphorylate several residues in the non-helical tail regions of myosin IIB and myosin IIC to control myosin II filamentation. MHC undergoes phosphorylation on T1823, T1833, and T2029 residues in the tail region of myosin II in *Dictyostelium* (De la Roche et al., 2002). Phosphorylation of these sites controls myosin II filamentation and plays critical roles in regulating growth and development of *Dictyostelium*. Except vWKa kinase, all other identified alpha-kinase family members including MHCK-A, MHCK-B, MHCK-C, and MHCK-D are involved in phosphorylating these sites in *Dictyostelium* (Egelhoff et al., 2005; Yumura et al., 2005; Underwood et al., 2010). Although vWKa does not directly phosphorylate MHC *in vitro* but regulates myosin II expression and filamentation in cells by unknown mechanism (Betapudi et al., 2005). Unlike other alpha kinases involved in regulating myosin II, vWKa displays specific sub-cellular localization to contractile vacuoles that are known to expel toxic metals and excess water from the cytoplasm of amoeba. Though the underlying mechanisms are yet to be uncovered, the myosin II-mediated mechanical work has been implicated in regulating the dynamics of contractile vacuoles and survival of *Dictyostelium discoideum* in abnormal osmotic conditions (Betapudi and Egelhoff, 2009). vWKa regulates myosin II expression and filament disassembly by unknown mechanisms to protect amoeba from osmotic shock death (Betapudi and Egelhoff, 2009). Phosphatases specific to the heavy chains of myosin II motor proteins are yet to be identified in mammals.

Many proteins including S100A4, lethal giant larvae (Lgl), myosin binding protein H, and S100P bind MHCs to control phosphorylation and filament assembly of myosin II in flies and mammals (Kriajevska et al., 1994; Ford et al., 1997; Vasioukhin, 2006; Du et al., 2012; Hosono et al., 2012). Lgl is a tumor suppressor protein and forms a complex with C-terminal ends of the MHC of myosin II to control cell proliferation. However, the Lgl-myosin II complex dissociates when myosin II heavy chain is phosphorylated by PKC (Strand et al., 1994; Kalmes et al., 1996; Plant et al., 2003; Betschinger et al., 2005). Lgl binds coiled-coil regions of the MHC to control myosin II filamentation and localization (De et al., 1999; Dahan et al., 2012). Deletion of the Lgl located specific region in the human chromosome 17 has been implicated in the development of Smith-Magenis Syndrome, a developmental disorder that affects many body parts, intellectual disability, and sleep disturbances (Smith et al., 1986; Koyama et al., 1996; De et al., 2001). However, role of mutated Lgl in controlling myosin II phosphorylation and cellular functions remains elusive. The metastasis factor mts1 also called S100A4 or calvasculin, a member of the S100 family of calcium-binding proteins, binds C-terminal ends of the MHC of myosin II. Binding of S100A4 to C-terminal ends of the MHC promotes phosphorylation on S1943 and disassembly of myosin II filamentation; however, the underlying mechanisms remain unknown to date (Li et al., 2003; Badyal et al., 2011; Mitsuhashi et al., 2011; Kiss et al., 2012). S100-P, another member of S100 family of calcium-binding proteins and a novel therapeutic target for cancer, interacts with myosin II in cells. S100-P has been implicated in controlling myosin II filamentation and cell migration (Du et al., 2012). Myosin binding protein H (MYBPH) binds ROCK1 to control RLC phosphorylation and cell migration. MYBPH also binds MHC to control myosin II filamentation and cell migration; however, the underlying mechanisms are not clearly understood (Hosono et al., 2012). Recent studies suggest that the unassembled myosin II with phosphorylated RLC plays a role in the initiation of focal adhesion complexes formation and cell membrane extension (Shutova et al., 2012). It would be interesting to understand the coordinated regulation of RLC and MCH phosphorylation adopted by cell to regulate myosin II filamentation and cellular functions. Though the underlying mechanisms are not clearly understood, Tropomyosin, an integral part of the actin cytoskeleton system in cells has been implicated in regulating myosin II localization to plasma membrane and stress fiber formation (Bryce et al., 2003). Myosin II activity is also controlled by Supervillin, an actin filament binding and cell membrane associated scaffolding protein. Supervillin binds MLCK to control RLC phosphorylation and myosin II activity (Takizawa et al., 2007). Thus, non-muscle myosin II motor proteins are regulated by several proteins at multiple levels to perform dedicated cellular functions.

## Myosin II motor proteins in predisposing humans to diseases

Plants live normal life without class II myosins but mammals require these multifunctional molecular machines for survival and growth. Because the *MYH9* germline-ablated mice without myosin IIA die on 6.5 embryonic day (E) due to defective cell-cell interaction and lack of polarized visceral endoderm (Conti et al., 2004). The *MYH10* germline-ablated mice with no myosin IIB survive till E14.5 and then die due to brain and cardiac developmental defects (Tullio et al., 1997, 2001). However, the *MYH14*-ablated mice in the absence of myosin IIC can survive with no obvious defects till adulthood but require the expression of myosin IIB (Ma et al., 2010). Misregulation, mutations, and alternative splicing of *MYH9, MY10*, and *MY14* predispose humans to the onset and progression of many diseases (Table 1). More than 45 mutations are identified in *MYH9* to date and some of them are linked to a large number of autosomal-dominant disorders including May-Hegglin anomaly, Sebastian platelet syndrome, Fetchner syndrome, Bernard-Soulier syndrome, Alport syndrome, and Epstein syndrome. These diseases are collectively called *MYH9*-related diseases (MYH9RD) (Kelley et al., 2000; Burt et al., 2008; Pecci et al., 2008; Balduini et al., 2011). The MYH9RD patients with mutations in the motor domain (R702C/H and R1165C/L) of myosin IIA develop deafness, cataract, Döhle-like inclusions, nephritis, and thrombocytopenia with enlarged platelets in their middle age (Pecci et al., 2008, 2014; De et al., 2013). An estimated 30–70 percent of MYH9RD patients develop kidney disease in their early adulthood. Leukocytes of the MYH9RD patients carry non-functional myosin IIA clumps. However, patients carrying mutations in the tail domain of myosin IIA (D1424H/N/Y, V1516M, E1841K, R1933X) show no symptoms of clinical relevance (Pecci et al., 2010). The overexpression of myosin IIA is implicated in causing enhanced cancer cell migration and metastasis as well as lung and kidney tumor invasion (Gupton and Waterman-Storer, 2006; Derycke et al., 2011; Xia et al., 2012). However, this hypothesis is downplayed by recent reports on myosin IIA roles in the posttranscriptional stabilization of p53 activity and repression of squamous cell carcinoma in mice (Schramek et al., 2014). A chimeric *MYH9-Alk* transcript formed by the fusion of *MYH9* and *ALK* (anaplastic lymphoma kinase) was observed in anaplastic large cell lymphoma but its disease relevance is yet to be established (Lamant et al., 2003). No mutation in *MYH10* that is relevant to a disease with any clinical symptom is reported to date; however, recently an E908X de novo mutation is reported in patients with microcephaly, hydrocephalus, cerebral, and cerebellar atrophy. An indirect link in between the expression of myosin IIB and progression of several diseases including, megakaryopoiesis, myocardial infarction, scar tissue formation, demyelination, and juvenile-onset neuronal ceroid lipofuscinosis (JCNL) or Batten disease is established (Antony-Debre et al., 2012). Batten disease, a lysosomal storage disorder is caused by mutations in *CLN3* that encodes a lysosomal membrane binding chaperone known to interact directly with myosin IIB. Mutations in *CLN3* are believed to affect interaction with myosin IIB as well as retrograde and anterograde trafficking in the Golgi complexes (Getty et al., 2011). Patients carrying CLN3 mutations show symptoms of seizures, psychomotor disturbances, dementia, and loss of vision (Cotman and Staropoli, 2012). Patients carrying mutations in *MYH14* are also linked to many diseases including hereditary blindness (DFNA4), hoarseness, peripheral neuropathy, and myopathy (Donaudy et al., 2004; Choi et al., 2011). In addition, patients expressing aberrant splicing products of *MYH14* develop myotonic dystrophy type 1 (DM1), a progressive multisystem genetic disorder that affects 1 in 8000 people worldwide (Rinaldi et al., 2012; Kumar et al., 2013).

**Table 1:** **Defects and associated diseases of myosin II motor proteins and their regulators**.

**Gene**	**Mutation/Defect**	**Disease**	**Common symptoms**
*MYH9*	R702C/H R1165C/L and many^*^	MYH9RD (May-Hegglin anomaly, Sebastian platelet syndrome, Fetchner, Bernard-Soulier syndrome, Alport syndrome, Epstein syndrome)	Thrombocytopenia, enlarged platelets, deafness, cataract, nephritis, and Döhle-like inclusions.
	*MYH9-Alk* chimeric Transcript^**^	Anaplastic large cell lymphoma	Blood cancer, painless swelling of lymph nodes, and rapid weight loss.
	Overexpression^$^	Cancer metastasis	–
*MYH10*	E908X (*de novo*)	Microcephaly, hydrocephalus, cerebral and cerebellar atrophy	Small head, dwarfism or short stature, delayed motor, and speech functions.
	Downregulation	Megakaryopoiesis, myocardial infarction, demyelination, Batten disease	Chest pain, dizziness, nausea, ocular paralysis, speech problem, and impaired vision.
*MYH14*	S7X, S120L, G376C, R726S, L976F	Hereditary blindness, hearing impairment (DFNA4), peripheral neuropathy, myopathy, hoarseness	Deafness, loss of vision, burning pain, numbness, changes in skin, hair or nail, dizziness, and paralysis.
	Aberrant splicing	Myotonic dystrophy type 1 (DM1) or Steinert disease	Weakness.
*ROCK*	Overexpression^$^	Cancer metastasis	–
*Mts1*	Overexpression^$^	Cancer metastasis	–
*Dmlc2^#^*	Δ2–46, S66A, S67A	Impairment of courtship	Inability of a fly to sing a courtship song.
*MYLK*	G601E	Cancer	–
	P147S	Asthma	–
	SNP	Asthma, acute lung injury, sepsis	–
*CLN3^@^*	L101P, L170P, Y199X, Q295K	Seizures, dementia, and psychomotor disturbances	Loss of vision and memory, mood swings, poor judgment.
*TRPM6*	R56X, S141L, R484X, S590X, Δ427–429, Δ736–737, Δ1260–1280	Seizures, Hypocalcemia, tetany, hypomagnesemia	Abnormal eye movement, convulsions, fatigue, numbness, anxiety, depression, dementia.
*LLgl*	Δ17p11.2^^^	Smith-Magenis Syndrome	Intellectual disability, sleep disturbances, behavior problems, defects in many body parts.

* *Refer Burt et al. (2008)*,

** *Found in the lymphocytes of lymphoma patients*,

$ *Implicated*,

# *Encodes RLC in Drosophila*,

@ *Interacts directly with myosin IIB, Δ deletion, SNP, single nucleotide polymorphism*;

∧ *LLgl, located region in chromosome 17*.

In addition, the overexpression of myosin II upstream regulators ROCK and Mts1 is implicated in spreading cancer (Sandquist et al., 2006; Boye and Maelandsmo, 2010; Kim and Adelstein, 2011). Mutations in RLC were shown to affect singing male courtship song in flies (Chakravorty et al., 2014). Mutations in RLC phosphorylating *MYLK* are linked to cancer (Greenman et al., 2007) and familial aortic dissections that may cause sudden death (Wang et al., 2010). A few race-specific single nucleotide polymorphism variants of *MYLK* are linked to asthma, acute lung injury and sepsis (Gao et al., 2006, 2007; Flores et al., 2007). Hypomagnesemia patients with secondary hypocalcemia carry mutations in *TRPM6* that is known to regulate MHC phosphorylation (Schlingmann et al., 2002; Walder et al., 2002). Though the underlying mechanisms are not clearly understood, myosin II motor proteins are believed to be hijacked by many pathogens such as herpes simplex virus type 1 for egression (van et al., 2002; Arii et al., 2010), murine leukemia virus for infection (Lehmann et al., 2005), and *Salmonella* bacteria for growth in macrophages (Wasylnka et al., 2008). Kaposi's sarcoma herpes simplex virus that is known to cause AIDS related neoplasm manipulates c-Cbl and myosin II-mediated signaling pathway to induce macropinocytosis in order to infect blood vessels (Sharma-Walia et al., 2010). Some pathogens like HIV-1 selectively down-regulates myosin IIA in kidney and cause renal disease probably to escape clearance through urine (Hays et al., 2012). Dengue virus type 2 activates Rac1 and Cdc42-mediated signaling pathway to regulate myosin II for successful infection of host cells (Zamudio-Meza et al., 2009). Respiratory Syncytial Virus (RSV) that is known to cause severe respiratory tract infections is believed to activate actomyosin system for improved pathogenesis (Krzyzaniak et al., 2013). Pathogens like hepatitis C virus induce development of autoantibodies having binding affinity for myosin IIA perhaps as a part of escape strategy from host defense network (von Muhlen et al., 1995).

## Conclusion and perspectives

Molecular motor proteins are largely accepted as the most efficient transducers of cellular free energy into biological work that is critical for the sustenance of life. Class II myosins especially non-muscle myosin IIA, myosin IIB, and myosin IIC motor proteins are emerged as the main mechanotransducers of cellular-free energy that is necessary for driving multiple biological processes ranging from birth to death in mammals' life. During the past two decades, research on myosin II motor proteins was focused on understanding the underlying mechanisms of myosin II-mediated mechanotransduction in many biological systems. It is also proven beyond reasonable doubt that murine life does not exist without the expression of non-muscle myosin II motor proteins (Conti and Adelstein, 2008). Interestingly, many patients with mutated myosin IIA, myosin IIB, and myosin IIC paralogs are reported but none without these biological nanomachines to date. The extrapolation of these findings with caution may suggest that life in mammals does not exist without the expression of non-muscle myosin II motor proteins. Therefore, the emergence of genes that encode non-muscle myosin II motor proteins perhaps is a turning point in the evolution of mammals. During this process, humans acquired three different genes *Myh10, Myh11*, and *Myh14* with a significant homology in nucleotide sequence. It is generally believed that humans do require the expression of all three functional non-muscle myosin II motor proteins to maintain normal growth, development, and disease resistance. But why human cell and tissue types display differential expression of myosin II paralogs still remains unanswered. Part of the reasons could be due to their specialization in mediating dedicated functions that are specific to each cell and tissue type. However, this concept will benefit from further understanding of structural and posttranslational modifications of all three different myosin II complexes. Although we made progress in identifying several mutations in myosin II motors proteins and their regulating proteins, very little is known about the disease-relevant mutations in myosin II motor proteins. Novel strategies for management and diagnosis of MYH9RD patients are required (Althaus and Greinacher, 2010). This area of research requires additional attention to gain more insights for the development of myosin II-based novel therapeutic approaches in future. Many modern cell biologists recognize myosin II motor proteins as key drivers of cell migration and cytokinesis that are known to go awry in cancer and other pathological conditions. Although overexpression of myosin II motor proteins has been implicated in driving cancer progression and metastasis, further understanding of their specific expression profiles in every cancer type will help designing therapeutic developments. Also, expanding our limited knowledge on the expression of chimeric as well as alternate splicing products of non-muscle myosin II motor proteins in pathological conditions will allow development of treatment options. During the past two decades, we made a very limited progress on understanding how pathogens hijack non-muscle myosin II motor proteins for their efficient infection and propagation. Understanding what made these dedicated molecular machines to work for the interests of pathogens is no less than a challenge to cell biologists in future. We are yet to understand how myosin II motor proteins mediate release of microvesicles that are known to make inter cellular communications and promote progression of many human diseases. Myosin II-mediated mechanotransduction has been implicated in the regulation of stem cell proliferation and differentiation (Chen et al., 2014). Additional efforts to understand the mechanical roles of myosin IIA, IIB, and IIC motor proteins will have a significant impact on stem cells-based tissue engineering, synthetic bioengineering, and therapeutic development.

### Conflict of interest statement

The author declares that the research was conducted in the absence of any commercial or financial relationships that could be construed as a potential conflict of interest.
